# Development of a Sex-Specific Risk Scoring System for the Prediction of Cognitively Normal People to Patients With Mild Cognitive Impairment (SRSS-CNMCI)

**DOI:** 10.3389/fnagi.2021.774804

**Published:** 2022-01-25

**Authors:** Wen Luo, Hao Wen, Shuqi Ge, Chunzhi Tang, Xiufeng Liu, Liming Lu

**Affiliations:** ^1^School of Medical Information Engineering, Guangzhou University of Chinese Medicine, Guangzhou, China; ^2^Evidence-Based Medicine and Data Science Centre, Guangzhou University of Chinese Medicine, Guangzhou, China; ^3^Department of Neurology, The Sun Yat-sen Memorial Hospital of Sun Yat-sen University, Guangzhou, China; ^4^South China Research Center for Acupuncture and Moxibustion, Medical College of Acu-Moxi and Rehabilitation, Guangzhou University of Chinese Medicine, Guangzhou, China

**Keywords:** sex-specific, scoring system, conversion, cognitively normal (CN), mild cognitive impairment (MCI), Alzheimer’s disease (AD)

## Abstract

**Objective:**

We aimed to develop a sex-specific risk scoring system, abbreviated as SRSS-CNMCI, for the prediction of the conversion of cognitively normal (CN) people into patients with Mild Cognitive Impairment (MCI) to provide a reliable tool for the prevention of MCI.

**Methods:**

CN at baseline participants 61–90 years of age were selected from the Alzheimer’s Disease Neuroimaging Initiative (ADNI) database with at least one follow-up. Multivariable Cox proportional hazards models were used to identify the major risk factors associated with the conversion from CN to MCI and to develop the SRSS-CNMCI. Receiver operating characteristic (ROC) curve analysis was used to determine risk cutoff points corresponding to an optimal prediction. The results were externally validated, including evaluation of the discrimination and calibration in the Harvard Aging Brain Study (HABS) database.

**Results:**

A total of 471 participants, including 240 female (51%) and 231 male participants (49%) aged from 61 to 90 years, were included in the study cohort. The final multivariable models and the SRSS-CNMCI included age, *APOE e4*, mini mental state examination (MMSE) and clinical dementia rating (CDR). The *C*-statistics of the SRSS-CNMCI were 0.902 in the female subgroup and 0.911 in the male subgroup. The cutoff point of high and low risks was 33% in the female subgroup, indicating that more than 33% female participants were considered to have a high risk, and more than 9% participants were considered to have a high risk in the male subgroup. The SRSS-CNMCI performed well in the external cohort: the *C*-statistics were 0.950 in the female subgroup and 0.965 in the male subgroup.

**Conclusion:**

The SRSS-CNMCI performs well in various cohorts and provides an accurate prediction and a generalization.

## Introduction

Alzheimer’s disease (AD) is a neurodegenerative disease that progresses over time (Alzheimer’s disease facts and figures, 2020). The progression of AD includes three stages: preclinical Alzheimer’s disease, Mild Cognitive Impairment (MCI) due to Alzheimer’s disease and dementia due to Alzheimer’s disease (Albert et al., 2011; McKhann et al., 2011; Sperling et al., 2011).

According to the latest Alzheimer’s disease report 2020, the number of people aged 65 years and older with AD is projected to reach 152 million worldwide by 2050 (Alzheimer’s disease facts and figures, 2020). The total annual payments for health care and long-term care for patients with AD have been approximately $305 billion in 2020 and were estimated to be more than $1.1 trillion in 2050 (Alzheimer’s disease facts and figures, 2020), which may impose an enormous financial burden on patient families and society. In China, it is estimated that the number of AD patients in China is expected to rise to more than 16 million by 2030 (Bo et al., 2019; Bai and Dong, 2021). Jia et al. (2018) estimated that the annual total costs are predicted to reach $507.49 billion in 2030 and $1.89 trillion in 2050, and the global estimates of costs for AD will be $9.12 trillion in 2050 basing on this results, much more than the predictions by the Alzheimer’s disease report 2020. Therefore China bears a heavy burden of AD costs, which greatly change the estimates of AD cost worldwide. Korea is one of the fastest aging countries worldwide, estimating that the prevalence of AD in Korea in 2020 was 10.25% among the people over 65 years old, and it will increase to about 15.91% by 2050. Between 2015 and 2019, The total health-economic cost of AD increased by about 1.5 times in the last 5 years and was estimated to be about $4218 million (Shon and Yoon, 2021). In Japan, the numbers of people living with AD in 2025 and 2060 were estimated to be approximately 6.5 to 7 million and 8.5 to 11.5 million, respectively (Ikeda et al., 2021). The societal costs of AD was projected to reach JPY 24.3 trillion ($188.9 billion) by 2060 (Ohno et al., 2021). According to the above data, AD has become a central public health issue in China, South Korea, Japan and other East Asian countries and even the whole world.

Studies have shown that 15% of MCI patients over 65 years of age develop AD after 2 years of follow-up (Petersen et al., 2018); 32% of MCI patients develop AD during 5 years of follow-up (Ward et al., 2013), and 38% of MCI patients develop AD after 5 years of follow-up (Mitchell and Shiri-Feshki, 2009). Thus, patients with MCI have a high risk of rapid conversion to AD. We believe that prevention of the development of MCI in normal people requires more attention. Therefore, the development of a predictive model may be useful for identification and prediction of appearance of the clinical symptoms or mild cognitive impairment to make sure that people receive an early treatment for AD prevention.

Steenland et al. (2018) developed a “Framingham-like” prediction model to predict the progression from unimpaired cognition to amnestic mild cognitive impairment (aMCI) using several dichotomous risk factors, including the memory summary score, characteristics of the hippocampus and the Tau/Aβ ratio. The *C*-statistic of this model was 0.80, and the data sample was not divided into a training set and test set; thus, the model lacked a validation. All risk factors were classified into four or two groups based on quartiles or ROC analysis, and the results indicated that the risk factor group could have lacked clinical significance. Barnes et al. (2014) used a Cox proportional hazards model to determine the risk factors influencing the AD progression and developed a point score ranging from 0 to 9, which used bootstrapping techniques to internally validate the model that lacked an external verification.

Sex is recognized as one of inherently important characteristics that influence the progression of AD (Santabárbara et al., 2019; Vermunt et al., 2019). Fisher et al. (2018) thought that advanced age and female sex are the two major non-modifiable risk factors for AD. The biological basis of the sex-based differences in AD onset and progression remain elusive, but two-thirds of clinically diagnosed cases of AD are women (Pellegrini et al., 2021). Oliveira et al. (2016) demonstrated that risk factors related to the onset of cognitive decline, including the history of coronary heart disease, age of AD onset and years of schooling, were different between male and female participants. Physiological characteristics, social status and other factors different in men and women were also different in patients with MCI. Therefore, we aimed to develop a sex-specific risk scoring system for the prediction of the conversion from cognitively normal people to patients with mild cognitive impairment (SRSS-CNMCI) to provide a reliable tool for the prevention of MCI. We performed external validation by using a new database to verify the generalization ability of the SRSS-CNMCI.

## Materials and Methods

### Data Sources and Participants

The present study used the data on the participants from two independent cohorts: the Alzheimer’s Disease Neuroimaging Initiative (ADNI) database^1^ for modeling and the Harvard Aging Brain Study (HABS) database^2^ for external validation.

In the ADNI, follow-up visits were carried out at 6-month intervals either in person or by telephone contact as required by the protocols (detailed protocols and associated documents are available at^3^). A complete battery of clinical and neuropsychological measures was collected at each time point. The initial diagnosis of participants at each time point is available in the table ‘‘DXSUM_PDXCONV_ADNIALL’’ (please see^4^ for further details).

Participants from the ADNI were included in the present study if they were (1) diagnosed as cognitively normal at baseline, (2) diagnosed with CN at all follow-up visits and classified as the non-converted group, (3) diagnosed with MCI at a certain follow-up visit after the baseline and thus classified as the converted group, and (4) 61–90 years of age. The calculation of the conversion time in the non-converted group was based on the follow-up duration of all follow-up visits in the study, and the conversion time in the converted group corresponded to the follow-up duration from the baseline to the first diagnosis of MCI.

Exclusion criteria included participants who (1) only had the baseline data and (2) were converted to AD directly.

Participants in the HABS database were selected for external validation according to the same inclusion and exclusion criteria used to select ADNI participants.

### Informed Consent

For the ADNI, each participant gave written informed consent for imaging and neuropsychological testing in accordance with the Human Subjects Research Committee Guidelines. Please see www.adni-info.org for further details. All participants in the HABS provided a written informed consent before the procedures of the study.

### Statistical Analysis

At baseline, continuous variables were presented as the mean ± standard deviation, and categorical variables were presented as quantity (percentage). Standardized difference values were used to determine the differences of all variables between the male and female groups, and standardized difference values greater than 0.1 corresponded to variables that were imbalanced between these two groups (Dongsheng and Dalton, 2012). For continuous baseline variable, the equation was as follows: $\mathrm{Standardized}\,\mathrm{Difference}=\frac{\left(\bar{{\text{x}}_{1}}-\bar{{\text{x}}_{2}}\right)}{\sqrt{\frac{{\text{s}}_{1}^{2}+{\text{s}}_{2}^{2}}{2}}},$ where x_1_ and x_2_ denote the sample mean of a baseline variable in each group, and s_1_ and s_2_ denote the sample variances, respectively. For categorical baseline variable, the equation was as follows: $\mathrm{Standardized}\,\mathrm{Difference}=\frac{\left({\text{p}}_{1}-{\text{p}}_{2}\right)}{\sqrt{\frac{{\text{p}}_{1}\,\left(1-{\text{p}}_{1}\right)+{\text{p}}_{2}\,\left(1-{\text{p}}_{2}\right)}{2}}},$ where p_1_ and p_2_ denote the proportion of a binary baseline variable in each group, respectively.

Risk factors were selected as described in previous reports (Stewart et al., 2009; Debette et al., 2011; Rönnemaa et al., 2011; Yang et al., 2011; Joas et al., 2012; Loy et al., 2014; Ben Bouallègue et al., 2017; Donohue et al., 2017; Gottesman et al., 2017; Abell et al., 2018; Alzheimer’s disease facts and figures, 2020; Guan et al., 2020; Suzuki et al., 2020; Kuang et al., 2021), including age, race, years of education, apolipoprotein E allele *e4* (*APOE e4*), family history of dementia (FHD), mini mental state examination (MMSE), the clinical dementia rating (CDR), systolic blood pressure and diastolic blood pressure.

The ADNI was a longitudinal cohort study that collected the data and recorded the time and diagnosis at each follow-up visit. Therefore, we developed a sex-specific risk scoring system (SRSS-CNMCI) based on a multivariable Cox proportional hazards model. Two dependent variables, including the conversion situation and conversion time, were incorporated in the model. Age was forcibly included in the multivariable models as one of independent variables because previous studies have shown that age is the greatest risk factor (Alzheimer’s disease facts figures, 2020).

The method of the development of the SRSS-CNMCI was based on a multivariable Cox proportional hazards model as follows. Step 1: Cox proportional hazards models for each risk factor yielded the corresponding regression coefficients (β_*i*_). Step 2: All risk factors were subgrouped according to clinical significance, and the median values for all subgroups were selected as the reference values (*W*_*i*_) of the subgroups. Step 3: For each risk factor, the first subgroup of the reference values (*W*_*i*_) was selected as the basic risk reference value (*W*_*iREF*_) for this factor. Step 4: The differences (*D*_*i*_) from the corresponding basic risk reference values (*W*_*iREF*_) were calculated based on the regression coefficients (β_*i*_) and reference values (*W*_*i*_). The equation was as follows: $D_{i}\,=\beta_{i}^{*}\,\,\left(W_{i}-W_{i\,R\,E\,F}\right)$. Step 5: A constant (*B*) was used to represent a single score. Step 6: The scores corresponding to all subgroups of risk factors (*P*_*i*_) were calculated based on *D*_*i*_ and *B*. The equation was as follows: *P*_*i*_ = *D*_*i*_ / *B*. Step 7: The absolute risk assigned to the total score was calculated according to the variation of the Cox regression defined by the equation: ($\overset{\wedge }{P}=1-S_{0}\,{\left(t\right)}^{\exp\left(\beta_{a}^{*}\,W_{a}+B^{*}\,\mathrm{total}\,\_\,\mathrm{point}-\sum \beta_{i^{*}}\,M_{i}\right)}$), where *S_0_(t)* is the average survival rate of participants in the ADNI at t years estimated by Kaplan–Meier analysis, and *M*_*i*_ is the mean or proportion of the risk factor.

Receiver operating characteristic (ROC) curve analysis was used to determine the risk probability cutoff points corresponding to the optimal prediction effect (Shaw et al., 2009). The risk probability exceeding this cutoff point was considered a high risk. The cutoff points were calculated as the maximum Youden’s index.

Model discrimination was calculated as the *C*-statistics similar to the area under the receiver operating characteristic curve (AUC; Nam, 2000), which represents an estimate of risk probability corresponding to assignment of a higher risk by the model to participants who converted to MCI versus those who did not convert to MCI. The Hosmer–Lemeshow χ^2^ statistic was used to estimate the model calibration based on comparison of the differences between the predicted and actual event rates.

External validation included an evaluation of discrimination and calibration in the HABS. All analyses were performed using SPSS Statistics 22.0 and Python 3.7.4.

## Results

### Workflow of Selected Participants

**FIGURE 1:**
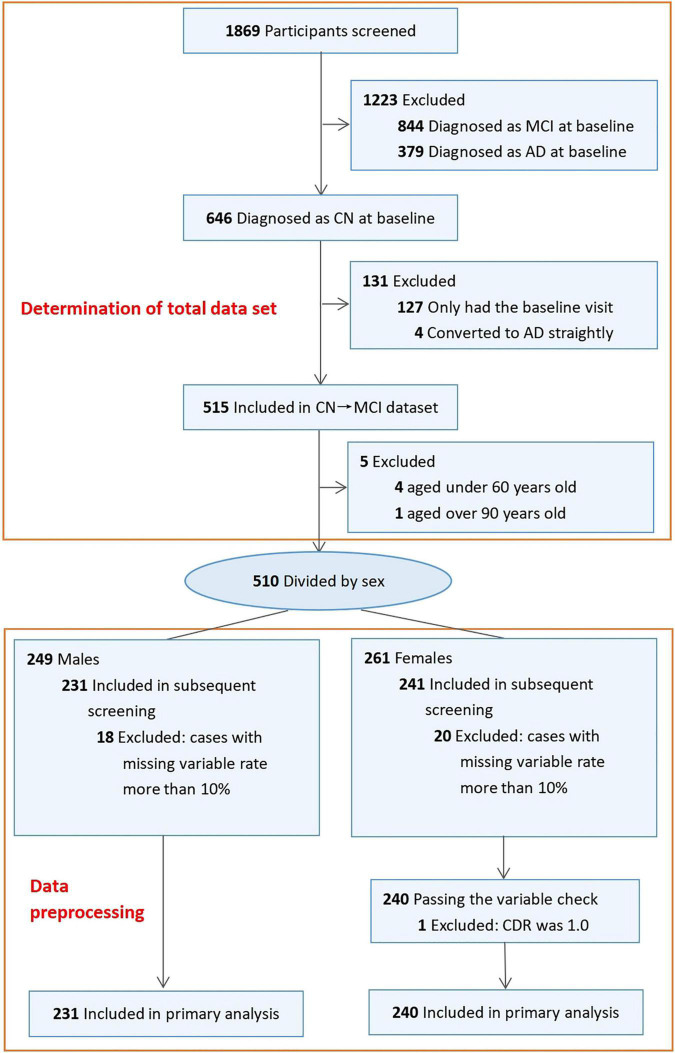
Workflow of selected participants in ADNI.

In the ADNI, 1869 participants were selected based on eligibility. The inclusion and exclusion criteria were used to finally select 510 participants, which were divided into two subgroups: male (*n* = 249) and female (*n* = 261). According to the requirements of data preprocessing, a total of 18 cases with missing variable rates greater than 10% were excluded from the male subgroup, and 20 cases were excluded from the female subgroup; a case with abnormal CDR values was excluded in the female subgroup. A total of 471 participants, including 240 females (51%) and 231 males (49%) aged from 61 to 90 years, were eventually included in the study cohort (Figure 1).

### Characteristics of Included Participants

In the female subgroup, participants with CN at baseline (*n* = 240) were monitored for the first event of the conversion to MCI for a maximum of 12 years between October 2005 and April 2019, and 37 participants converted to MCI during the follow-up (15%) with 3-year median conversion time (mean: 3.87, std: 2.68). In the male subgroup, participants with CN at baseline (*n* = 231) were monitored for the first event of conversion to MCI for a maximum of 12 years between October 2005 and February 2019, and 52 participants converted to MCI during the follow-up (23%) with 4-year median conversion time (mean: 3.77, std: 2.67).

Supplementary Table 1 provides a description of baseline demographic and clinical characteristics of participants of both sexes in the ADNI. In the male and female subgroups, older participants more easily converted to MCI, and participants who had fewer years of education more easily converted to MCI. Age, years of education, *APOE e4*, FHD, and MMSE score were imbalanced between the male and female subgroups (standardized difference >0.1). There were no significant differences between the male and female subgroups in race, systolic blood pressure, diastolic blood pressure or CDR score (standardized difference <0.1).

### Univariate Cox Regression Analysis Based on Sex

In the male and female subgroups, univariate Cox regression analysis was used to identify risk factors (Supplementary Table 2). Both MMSE and CDR scores were strongly correlated with the risk of the conversion to MCI (*P* < 0.001 for MMSE and CDR in the male subgroup; *P* < 0.001 for CDR and *P* = 0.003 for MMSE in the female subgroup). The proportion of *APOE e4* carriers (*P* = 0.019) was associated with the risk of the conversion to MCI in the female subgroup, and age (*P* = 0.019) was associated with the risk of the conversion to MCI in the male subgroup.

### Multivariable Cox Proportional Hazards Regression

The final multivariable models (Supplementary Tables 3A,B) and the SRSS-CNMCI (Tables 1a,b) included age, *APOE e4*, MMSE, and CDR. The models in the male and female subgroups based on age, *APOE e4*, MMSE, and CDR were statistically significant (*P* < 0.000).

**TABLE 1a:** The first part of the SRSS-CNMCIs – Risk scores.

Risk factor	Categories	Risk score (Female)	Risk score (Male)
Age	61–70	0	0
	71–80	2	2
	81–90	4	4
APOE e4 +	No	0	0
	Yes	2	3
MMSE	≤26	1	5
	>26	0	0
CDR	0.0	0	0
	0.5	14	9

*The scores are converted based on the Cox proportional hazard functions.*

*A category with a score of zero should not be misinterpreted as an indication of the presence of a biological threshold effect.*

*Minimum of the total score: 0.*

*Maximum of the total score: 23.*

**TABLE 1b:** The second part of the SRSS-CNMCIs – Predicted 12-year risk of converting to MCI assigned to the risk score.

Risk score (Female)	Risk score (Male)	Predicted risk
≤1	≤10	≤10%
2–8	11–15	11–20%
9–12	16–18	21–30%
13–16	19–21	31–40%
17–19	≥22	41–50%
≥20	–	>50%

*Maximum of Predicted Risk (Female): 65%.*

*Maximum of Predicted Risk (Male): 48%.*

### SRSS-CNMCI Development

**FIGURE 2:**
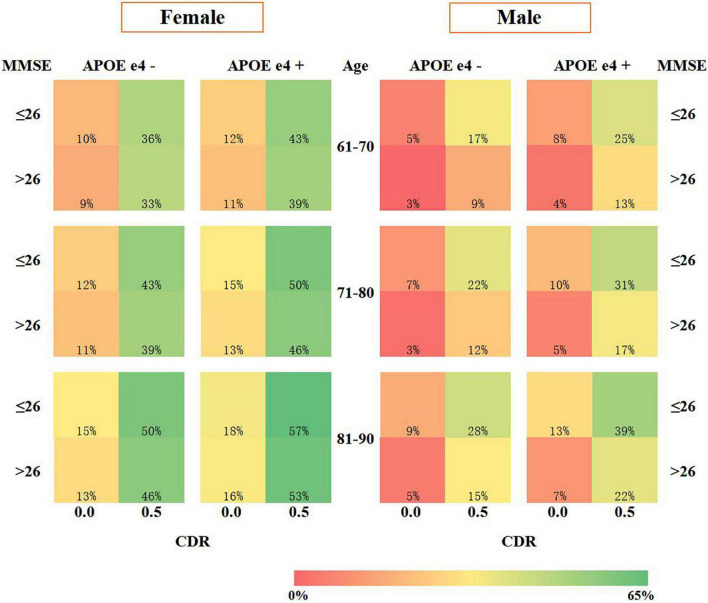
Risk prediction visualization of the SRSS-CNMCI.

We developed an SRSS-CNMCI for each risk factor (Table 1a) and assigned a total score to a specific absolute risk of the conversion to MCI in cognitively normal participants aged from 61 to 90 years (Table 1b). The SRSS-CNMCI yielded a range of total scores from 0 to 23 in the female and male subgroups; however, the risk scores for three risk factors, including *APOE e4*, MMSE, and CDR, were different between the male and female subgroups. The maximum predicted risk in the SRSS-CNMCI was 65% in the female subgroup and only 48% in the male subgroup. Figure 2 presents a heat map, which visualizes the risk of the conversion of cognitively normal people to MCI predicted by the SRSS-CNMCI based on various risk factors.

**FIGURE 3:**
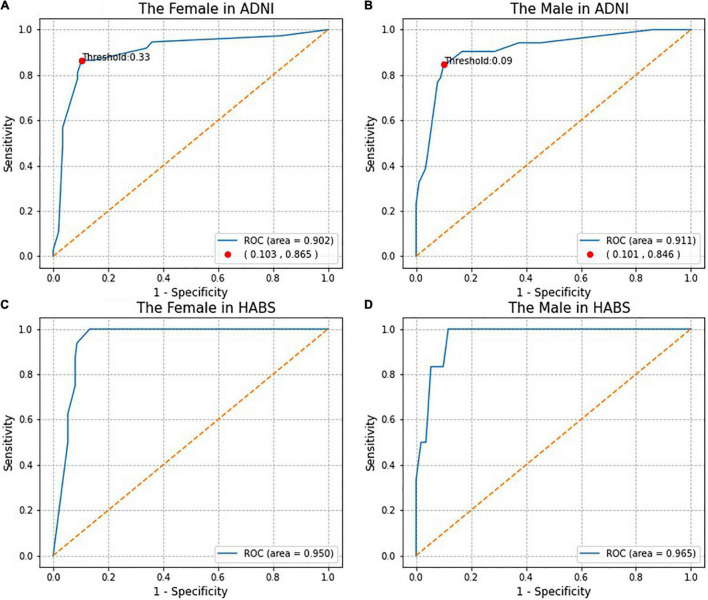
The Receiver operating characteristic (ROC) curves and the Marking of high and low risk thresholds.

Figure 3 shows the ROC curves for the SRSS-CNMCI. According to Figures 3A,B, the AUCs for the SRSS-CNMCI were 0.902 in the female subgroup and 0.911 in the male subgroup, indicating that the SRSS-CNMCI very well predicted the risk of the conversion to MCI. The risks of the conversion to MCI predicted by the SRSS-CNMCI were similar to observed risks (χ^2^ = 35.56, *P* = 0.154 in the female subgroup; χ^2^ = 45.0, *P* = 0.271 in the male subgroup).

**FIGURE 4:**
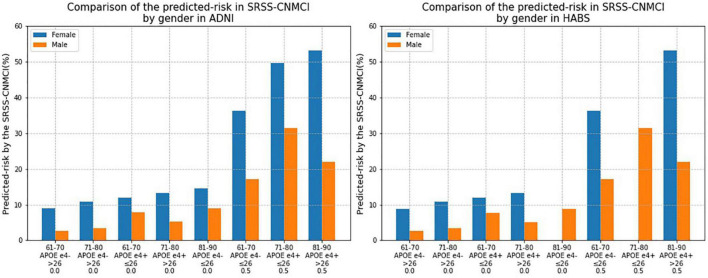
Comparison of the risk of converting to MCI calculated by the SRSS-CNMCI by sex in ADNI and HABS.

Comparison of predicted risks of the conversion to MCI according to the SRSS-CNMCI based on sex in the ADNI and HABS (Figure 4) intuitively suggested that the risks, which were predicted by the same risk factor combinations, were different between the male and female subgroups, and all risks in the female subgroups were higher than those in the male subgroups in the ADNI and HABS.

### Receiver Operating Characteristic Analysis

Receiver operating characteristic analysis of actual diagnostic risk of the conversion to MCI versus the risk of the conversion to MCI predicted by the SRSS-CNMCI provided a cutoff point for high and low risks at the greatest accuracy of the diagnostic test. The cutoff point for high and low risks was 33% in the female subgroup, indicating that more than 33% of female participants were considered a high risk, and more than 9% of participants were considered a high risk in the male subgroup (Figures 3A,B). The *C-*statistics indicated that dichotomized model based on high or low risk classifications performed well: the *C*-statistics were 0.881 in the female subgroup and 0.873 in the male subgroup.

**FIGURE 5:**
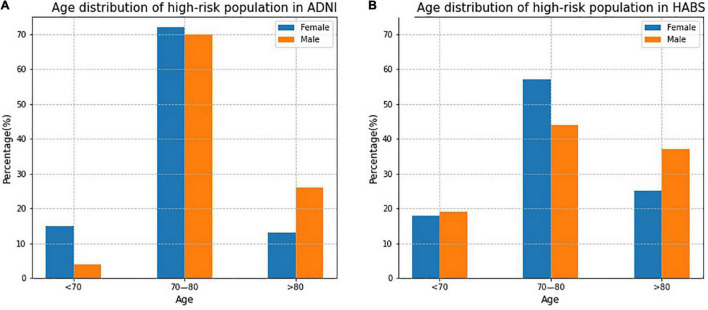
The age distribution of high-risk participants predicted by the SRSS-CNMCI in ADNI and HABS.

Figure 5 shows the age distribution of high-risk participants predicted by the SRSS-CNMCI in the ADNI and HABS. In both the ADNI and HABS, the age of high-risk participants was predominantly between 70 and 80 years, and the proportion of female participants was higher than that of male participants. The proportion of high-risk participants 70–80 years of age was as high as 70% in the female and male subgroups in the ADNI.

### Validation of SRSS-CNMCI

To evaluate the generalization performance of the SRSS-CNMCI in the HABS, a total of 283 participants, including 166 women (59%) and 117 men (41%) aged from 61 to 90 years, were selected based on the same inclusion and exclusion criteria used for the selection in the ADNI (eFigure 1 of Supplementary Material). As shown in Figures 3C,D, the AUCs for the SRSS-CNMCI were 0.950 in the female subgroup and 0.965 in the male subgroup, indicating that the SRSS-CNMCI performed very well in the HABS. The risks predicted by the SRSS-CNMCI were similar to the observed risks (χ^2^ = 30.0, *P* = 0.314 in the female subgroup; χ^2^ = 20.00, *P* = 0.220 in the male subgroup) in the HABS.

## Discussion

### Summary of the Results

We presented a sex-specific scoring system (SRSS-CNMCI) for the prediction of the risk of the conversion to MCI within 12 years in cognitively normal participants aged from 61 to 90 years. The SRSS-CNMCI estimated the absolute risk of the conversion and assessed the risk level, corresponding to high or low conversion risks of participants, which provided an intuitive understanding of the risk. The risk of the conversion to MCI in cognitive normal participants aged from 61 to 90 years predicted by the SRSS-CNMCI was significantly different between the female and male subgroups (*P* = 0.005 according to an independent *T*-test); the SRSS-CNMCI performed well in the ADNI and HABS, indicating that the studies of sex-specific models should be continued. This result also indicated that specific monitoring and treatment plans should be implemented in men and women, respectively.

### Study Strengths

First, previous studies on risk scoring and prediction models related to AD or MCI (Steenland et al., 2018; Zhang et al., 2018; Yue and Shifu, 2019) rarely consider sex-specific differences. However, the present study demonstrated a significant difference between predicted risks of the conversion to MCI in cognitively normal female and male participants according to the SRSS-CNMCI, which cannot be ignored. Second, most previous studies considered only the conversion to MCI, ignored the role of conversion time, and did not consider conversion time as one of dependent variables (Steenland et al., 2018). Therefore, we used a Cox proportional hazards model to develop the SRSS-CNMCI and used both the event of the conversion to MCI and conversion time as dependent variables. Third, we comprehensively evaluated the performance of the SRSS-CNMCI (D’Agostino et al., 2001), estimated the discrimination to evaluate the accuracy of the SRSS-CNMCI, and estimated the calibration to evaluate consistency of the predicted and the actual values. Fourth, some studies demonstrated that external validation of the risk function should be performed in new independent datasets (Schnabel et al., 2009). External validation was one of the methods used to evaluate the generalization ability of the SRSS-CNMCI; hence, we performed an external validation in an independent HABS. Evaluation of the discrimination and calibration (Figures 3C,D) demonstrated that the SRSS-CNMCI performed well in the HABS, indicating that the SRSS-CNMCI has a good generalization ability and can be extended to other cohorts. Fifth, we used ROC analysis to determine the cutoff for high and low risks predicted by the SRSS-CNMCI and demonstrated that dichotomized model, which involved high or low risk classifications calculated based on the cutoff, performed well (the *C*-statistics were 0.881 in the female subgroup and 0.873 in the male subgroup), indicating that the cutoff values are reliable.

### Possible Reasons for Sex Differences

Previous studies demonstrated significant sex-specific differences in the incidence rates and progression of AD and MCI (Hebert et al., 2013) primarily in the following aspects. First, in terms of brain structure, Pfefferbaum et al. (2013) demonstrated that in patients with MCI and AD, women manifested a faster decrease in the brain volume compared to men and men had higher brain reserves, indicating that men had a stronger ability to resist the disease than women. Second, in terms of hormones, studies on the effects of sex hormones on brain neurons demonstrated that sex hormones play a role in the entire life cycle of a person. Sex hormone levels and sexual genetic differences determine nerve regeneration in the brain and facilitate axon guidance in two-way development of the vessels and nerves, and the differences between men and women include the most notable features of sex hormones based on body type and variable expression levels (Rosario et al., 2011; Li et al., 2014; Filon et al., 2016; Chu et al., 2017). Third, in terms of social life, WookYoo et al. (2015) demonstrated that AD patients with better education presented with considerably lower damage of the structural connections of the brain than the general population, and the mean schooling was lower for females (Oliveira et al., 2016), which was similar to the findings of the present study. Our findings suggested that men had higher cognitive reserve than women in our sample and, as a result, were more resistant to neuropathological changes in the brain.

### Study Limitations

First, the risk factors included in the present study are not comprehensive. Our goal was to develop a simple and accurate predictive tool. Incorporation of the most common and easily accessible clinical indicators, such as body mass index (BMI) and daily activities (e.g., exercise frequency and reading time), to predict the risk of MCI conversion should be of greater value for early prevention. However, the study did not include sufficient number of samples to incorporate the data on height in the ADNI; hence, the BMI could not be calculated; furthermore, the data related to daily activities could not be obtained for the ADNI; however, some common variables mentioned above were not included in the development of the SRSS-CNMCI. Second, the sample size was not large enough. Although we used the world’s largest AD database (ADNI), we included only a small number of samples for model development. In the future, we will continue to expand the sample size and further improve the prediction performance of the SRSS-CNMCI.

### Consideration of Variable

Based on previous studies and clinical significance of risk factors, we purposefully incorporated clinical risk factors that are readily and routinely accessible in clinical trials and primary care. The present study included the data on demographic characteristics, genetics, cognitive tests, vital signs, and medical history and did not consider neuroimaging or Cerebrospinal Fluids (CSF) biomarkers. The majority of neuroimaging indices included in the prediction models describe the volume, surface area and thickness of a certain area of interest in the brain, such as middle temporal cortical thickness, hippocampal subcortical volume and right amygdala surface area (Barnes et al., 2014; Steenland et al., 2018; Fan and Cai, 2019), which lack relatively strong specific relationships with MCI. Considering high cost of neuroimaging analysis of patients and limited number of CSF biomarkers in the ADNI, we did not include neuroimaging and CSF biomarkers in the present study.

The multivariable Cox proportional hazards regression model was significant; *APOE e4* has the strongest impact on the risk of late-onset Alzheimer’s disease (Alzheimer’s disease facts and figures, 2020) and even though there was no significance in the model, *APOE e4* was still included in the present study. The multivariable Cox proportional hazards regression model yielded the same result in the male subgroup. The selection of risk factors in the present study was based on integration of clinical significance, the results of previous studies, and univariate and multivariate analyses. The difference in FHD was statistically significant only between men and women and had no effect on the conversion to MCI, which may be due to substantial recall bias and inaccurate collection of this information. Therefore, the role of FHD as a potential risk factor appears to be insufficiently convincing and does not justify its inclusion in subsequent studies.

## Conclusion

We successfully developed an SRSS-CNMCI prediction model with an accuracy of more than 90%, which can be used to accurately predict the conversion from CN to MCI.

## Data Availability Statement

The raw data supporting the conclusions of this article will be made available by the authors, without undue reservation.

## Ethics Statement

Ethical review and approval was not required for the study on human participants in accordance with the local legislation and institutional requirements. For the ADNI, each participant gave written informed consent for imaging and neuropsychological testing in accordance with the Human Subjects Research Committee Guidelines. Please see www.adni-info.org for further details. All participants in the HABS provided a written informed consent before the procedures of the study.

## Author Contributions

WL, XL, and LL: study concept and design. WL and SG: acquisition of data. WL and HW: analysis and interpretation of data. WL and LL: drafting of the manuscript. CT, XL, and LL: critical revision of the manuscript for important intellectual content. All authors contributed to the article and approved the submitted version.

## Conflict of Interest

The authors declare that the research was conducted in the absence of any commercial or financial relationships that could be construed as a potential conflict of interest.

## Publisher’s Note

All claims expressed in this article are solely those of the authors and do not necessarily represent those of their affiliated organizations, or those of the publisher, the editors and the reviewers. Any product that may be evaluated in this article, or claim that may be made by its manufacturer, is not guaranteed or endorsed by the publisher.

## Footnotes

1
http://adni.loni.usc.edu/

2
https://habs.mgh.harvard.edu/

3
http://adni.loni.usc.edu/methods/documents/

4
https://ida.loni.usc.edu/

## Abbreviations

SRSS-CNMCI: a sex-specific risk scoring system for the prediction of cognitively normal people to patients with mild cognitive impairment
CN: cognitively normal
MCI: mild cognitive impairment
ADNI: Alzheimer’s disease neuroimaging initiative
ROC: receiver operating characteristic curve
HABS: harvard aging brain study
MMSE: mini mental state examination
CDR: clinical dementia rating
AD: Alzheimer’s disease
aMCI: amnestic mild cognitive impairment
*APOE e4*: apolipoprotein E allele *e4*
FHD: the family history of dementia
AUC: area under the receiver operating characteristic (ROC) curve
BMI: body mass index
CSF: cerebrospinal fluids.
