# Supplementary material for: Development of a Sex-Specific Risk Scoring System for the Prediction of Cognitively Normal People to Patients With Mild Cognitive Impairment (SRSS-CNMCI)
Source: Front Aging Neurosci. 2022 Jan 25;13:774804. doi: 10.3389/fnagi.2021.774804 (PMC8823413; doi:10.3389/fnagi.2021.774804)

# Supplementary Material*

# Table of Contents

**Appendix 1: A supplementary description of the method**......2

**Database Introduction**......2

**Participants**......2

**Risk factors**......3

**Data preparation**......4

**References**......5

**Appendix 2: Baseline demographic and clinical characteristics by gender in ADNI**...... 7

**Appendix 3: Univariate Cox regression analysis by gender**......9

**Appendix 4: Multivariable Cox proportional hazards regression coefficients (adjusted for age) for the risk of converting to MCI**......10

**Appendix 5: Flow of screening participants in HABS**......11

* This supplementary material was provided by the authors to give readers further details on their article. The material was reviewed but not copyedited.

# Appendix 1: A supplementary description of the method

## Database Introduction

The ADNI was started in 2004 and is a multicenter project involving approximately 50 medical centers and universities in the United States and Canada to identify and track Alzheimer's disease early [49, 50]. The ADNI provides multidomain data, including demographics, neuroimaging, CSF biomarkers, genetics, cognitive tests and functional ability [14]. In ADNI, we downloaded data files in the format of .csv through different data columns under the "Study Data" module in the "Download" area.

HABS, a longitudinal observational study, aims to illuminate the earliest changes in molecular, functional and structural imaging markers that mark the conversion from cognitive normal to cognitive decline in preclinical Alzheimer's disease [51]. HABS data were obtained by filling in the research information and related data demand application through the webpage and receiving the data form via email after obtaining approval.

As longitudinal programs, participants in the ADNI and HABS were followed up at multiple time points each year.

## Participants

All participants in the ADNI provided written informed consent, and the criteria for inclusion and exclusion of participants are provided and approved by the ADNI website. Participants from the ADNI-1, ADNI-GO and ADNI-2 cohorts were included in the primary analysis.

Diagnostic data came from the following files compiled by ADNI, "DXSUM_PDXCONV_ADNIALL". The general inclusion/exclusion criteria for MCI participants in the ADNI are as follows: 1) a memory complaint; 2) MMSE scores between 24-30 (inclusive); 3) objective memory loss measured by education adjusted scores on Wechsler Memory Scale Logical Memory II; 4) a CDR of 0.5; 5) absence of significant levels of

impairment in other cognitive domains; 6) essentially preserved activities of daily living; and 7) an absence of dementia. Data from different domains derived from different files in the ADNI database were combined based on the participant ID and visit codes to form a total dataset.

## Risk factors

Numerous previous studies have shown that risk factors, such as older age [52,53], genetics [54,55], having a family history of Alzheimer's disease [56,57], education [58-60], systolic or diastolic blood pressure [22-25], having a family history of cardiovascular disease [1], and cognitive tests [14] are related to the onset and progression of AD.

We considered multidomain data, including demographic characteristics, genetics, cognitive tests, vital signs and medical history, all of which were of the domains of interest in this study.

For demographics, we considered age, years of education and race. Age and years of education were considered continuous variables, while race was classified as a categorical variable. Information on each risk factor was obtained from the "PTDEMOG" file.

For genetics, we focused on a family history of dementia (FHD) and apolipoprotein E genotype 4 (APOE e4). Researchers have identified several genes that increase the risk of AD, with the APOE e4 gene having the greatest effect on the risk of AD progression [1]. A recent study also showed that APOE is slightly specific in different clinical stages and is slightly exacerbated in late mild cognitive impairment (LMCI) [26]. Studies have shown that those who have a parent (first-degree relative) with AD are more likely to suffer from the disease than those who have no first-degree relative with AD [27]. The family history of dementia and APOE ε4 data were obtained from "FHQ" and "APOERES" files, respectively. Participants were identified as carriers of APOE ε4 upon registration and admission to the ADNI program [61].

For cognitive tests, the Mini-Mental State Examination (MMSE) and the Clinical Dementia Rating (CDR) were included in subsequent analysis. In previous studies of univariate analysis and multivariate modeling that affected the progress of AD and MCI, the most frequently used cognitive test indicators were the MMSE and CDR scores [26, 28-30]. In this study, the baseline MMSE and CDR scores of participants were used to describe baseline characteristics, while the

MMSE and CDR scores of the follow-up endpoint were used for modeling. The MMSE and CDR data were obtained from “MMSE” and “CDR” files, respectively.

For physical exams, we included systolic blood pressure and diastolic blood pressure. In studies exploring risk factors for cardiovascular disease, hypertension and prehypertension have been found to be associated with an increased risk of AD progression [22-25]. Longitudinal studies have also found that longer duration and/or higher or lower blood pressure are associated with the risk of AD onset and progression in old age [31-34]. In this study, the baseline systolic and diastolic blood pressure of participants were used to describe baseline characteristics, while the systolic and diastolic blood pressure of the follow-up endpoint were used for modeling. Data for physical exams were obtained from VITALS files.

## Data preparation

Data were collected from the ADNI database according to the inclusion and exclusion criteria, and the data files of different predictors were combined into a total dataset. There are two ways to combine a horizontal database with the file “DXSUM_PDXCONV_ADNIALL”, where the diagnostic data are located as the basic table: 1) demographic and other static data are merged with the base table through the participant ID (RID). 2) Dynamic data, such as vital signs and cognitive tests that changed over time, are combined with the diagnostic data table through two keywords, RID and the visit codes (VISCODE).

The combined dataset was preprocessed according to the following three steps. Step 1: Process missing values and check outliers. Participants with missing values of predictors were directly excluded if one of the following three conditions was met: 1) There is no quantitative correspondence between variables shown by previous studies, so it is impossible to infer the missing value of a variable from the value of one or more variables. 2) It is not clinically meaningful to infer the missing value of a certain predictor based on the value of several predictors or the value of multiple time points of the predictor itself. 3) Predictors with missing values accounted for more than 10%. Step 2: Unify data units, split data with the same name and different meanings, and merge data with different names and synonyms. In the case of multiple

units of quantitative data, select one of the most commonly used units and uniformly convert it to the value under this unit. Step 3: Re-encoding the value of the categorical variable. One of the most common values was selected as the basic value and was assigned a value of 0. The rest were assigned to other integers greater than zero according to the degree or other criteria.

Referring to previous studies, the original risk factors we selected included age, years of education, handedness, retirement status, years of retirement, family history of dementia, APOE e4 status, systolic blood pressure, diastolic blood pressure, history of cardiovascular disease, MMSE and CDR. To avoid being completely data-driven, the following three methods were used to screen the risk factors that were eventually included in the model: 1) according to previous studies, the risk factors that were clinically significant and had been proven to be associated with increased MCI risk were selected; 2) based on univariate analysis, risk factors with statistical significance ($P$<0.1) were preliminarily screened; and 3) based on multivariate analysis, risk factors with statistical significance ($P$<0.1) in the model were screened out.

## References


49. Weiner MW, Veitch DP. Introduction to special issue: Overview of Alzheimer's Disease Neuroimaging Initiative. Alzheimers Dement. 2015 Jul;11(7):730-3.

50. Petersen RC, Aisen PS, Beckett LA, Donohue MC, Gamst AC, Harvey DJ, Jack CR Jr, Jagust WJ, Shaw LM, Toga AW, Trojanowski JQ, Weiner MW. Alzheimer's Disease Neuroimaging Initiative (ADNI): clinical characterization. Neurology. 2010 Jan 19;74(3):201-9.

51. Dagley A, LaPoint M, Huijbers W, Hedden T, McLaren DG, Chatwal JP, Papp KV, Amariglio RE, Blacker D, Rentz DM, Johnson KA, Sperling RA, Schultz AP. Harvard Aging Brain Study: Dataset and accessibility. Neuroimage. 2017 Jan;144(Pt B):255-258.

52. Hebert LE, Bienias JL, Aggarwal NT, Wilson RS, Bennett DA, Shah RC, et al. Change in risk of Alzheimer disease over time. Neurology 2010;75:786-91.

53. Hebert LE, Weuve J, Scherr PA, Evans DA. Alzheimer disease in the United States (2010-2050) estimated using the 2010 Census. Neurology 2013;80(19):1778-83.

54. Saunders AM, Strittmatter WJ, Schmechel D, George-Hyslop PH, Pericak-VanceMA, Joo SH, et

al. Association of apolipoprotein E allele epsilon 4 with late-onset familial and sporadic Alzheimer’s disease. Neurology 1993;43:1467-72.

55. Farrer LA, Cupples LA, Haines JL, Hyman B, Kukull WA, Mayeux R, et al. Effects of age, sex, and ethnicity on the association between apolipoprotein E genotype and Alzheimer disease: A meta-analysis. JAMA 1997;278:1349-56.

56. Green RC, Cupples LA, Go R, Benke KS, Edeki T, Griffith PA, et al. Risk of dementia among white and African American relatives of patients with Alzheimer disease. JAMA 2002;287(3):329-36.

57. Mayeux R, Sano M, Chen J, Tatemichi T, Stern Y. Risk of dementia in first-degree relatives of patients with Alzheimer’s disease and related disorders. Arch Neurol 1991;48(3):269-73.

58. Stern Y. Cognitive reserve in ageing and Alzheimer’s disease. Lancet Neurol 2012;11(11):1006-12.

59. Sando SB, Melquist S, Cannon A, Hutton M, Sletvold O, Saltvedt I, et al. Risk-reducing effect of education in Alzheimer’s disease. Int J Geriatr Psychiatry 2008;23(11):1156-62.

60. Hendrie HC, Smith-Gamble V, Lane KA, Purnell C, Clark DO, Gao S. The Association of early life factors and declining incidence rates of dementia in an elderly population of African Americans. J Gerontol B Psychol Sci Soc Sci 2018;16(73, suppl 1):S82-9.

61. Trojanowski JQ, Vandeerstichele H, Korecka M, Clark CM, Aisen PS, Petersen RC, Blennow K, Soares H, Simon A, Lewczuk P, Dean R, Siemers E, Potter WZ, Weiner MW, Jack CR Jr, Jagust W, Toga AW, Lee VM, Shaw LM; Alzheimer's Disease Neuroimaging Initiative. Update on the biomarker core of the Alzheimer's Disease Neuroimaging Initiative subjects. Alzheimers Dement. 2010 May;6(3):230-8.

## Appendix 2: Baseline demographic and clinical characteristics based on sex in ADNI

**eTable 1. Baseline demographic and clinical characteristics based on sex in ADNI.**

| Risk factor | Female (n = 240) | | | Male (n = 231) | | | Standardized Difference |
|---|---|---|---|---|---|---|---|
| | Non-converted (n = 203) | Converted (n = 37) | All | Non-converted (n = 179) | Converted (n = 52) | All | |
| Age | 73.66±5.27 | 75.16±4.32 | 73.89±5.15 | 74.64±5.88 | 76.62±5.90 | 75.08±5.93 | 0.216 † |
| Race | | | | | | | |
| white | 185 (91.1) | 33 (89.2) | 218 (90.8) | 164 (91.6) | 47 (90.4) | 211 (91.3) | 0.018 |
| others | 18 (8.9) | 4 (10.8) | 22 (9.2) | 15 (8.4) | 5 (9.6) | 20 (8.7) | |
| Education (y) | 15.73±2.77 | 15.14±2.32 | 15.64±2.71 | 17.15±2.52 | 16.85±2.57 | 17.08±2.53 | 0.551 † |
| APOE e4 + | | | | | | | 0.117 † |
| no | 146 (71.9) | 19 (51.4) | 165 (68.8) | 133 (74.3) | 38 (73.1) | 171 (74.0) | |
| yes | 57 (28.1) | 18 (48.6) | 75 (31.3) | 46 (25.7) | 14 (26.9) | 60 (26.0) | |
| FHD | | | | | | | |
| no | 93 (45.8) | 16 (43.2) | 109 (45.4) | 102 (57.0) | 32 (61.5) | 134 (58.0) | 0.254 † |
| yes | 110 (54.2) | 21 (56.8) | 131 (54.6) | 77 (43.0) | 20 (38.5) | 97 (42.0) | |
| Systolic blood pressure | 133.71±17.65 | 135.30±17.78 | 133.96±17.64 | 132.54±15.55 | 132.77±14.81 | 132.59±15.36 | 0.083 |

| | | | | | | | |
|---|---|---|---|---|---|---|---|
| Diastolic blood pressure | 72.51±9.54 | 73.00±9.12 | 72.59±9.46 | 73.02±9.50 | 71.94±9.02 | 72.78±9.39 | 0.020 |
| MMSE | 29.07±1.16 | 29.35±0.72 | 29.12±1.11 | 29.07±1.10 | 28.73±1.29 | 28.99±1.15 | 0.111 † |
| CDR | | | | | | | |
| 0.0 | 203 (100) | 37 (100) | 240 (100) | 179 (100) | 52 (100) | 231 (100) | 0.000 |
| 0.5 | 0 | 0 | 0 | 0 | 0 | 0 | |
| Conversion time (y) | 3.78±2.67 | 4.35±2.76 | 3.87±2.68 | 3.90±2.69 | 3.35±2.60 | 3.77±2.67 | 0.034 |

Continuous variables were presented as the mean ± standard deviation, and categorical variables were presented as quantity (percentage).

†Standardized Difference $= \frac{(\overline{x_1}-\overline{x_2})}{\sqrt{\frac{s_1^2+s_2^2}{2}}}$ (continuous baseline variable), where $x_1$ and $x_2$ denote the sample mean of a baseline variable in each group, and $s_1$ and $s_2$ denote the sample variances, respectively.

Standardized Difference $= \frac{(p_1-p_2)}{\sqrt{\frac{p_1(1-p_1)+p_2(1-p_2)}{2}}}$ (categorical baseline variable), where $p_1$ and $p_2$ denote the proportion of a binary baseline variable in each group, respectively.

Measuring the difference between two groups.

Imbalance defined as absolute value greater than 0.10.

Abbreviations: FHD, family history of dementia; MMSE, the Mini-Mental State Examination score; CDR, the Clinical Dementia Rating score.

# Appendix 3: Univariate Cox regression analysis based on sex

**eTable 2. Univariate Cox regression analysis based on sex.**

| Risk factors | Female | | | Male | | |
|---|---|---|---|---|---|---|
| | **β coefficient** | **SE** | **P value** | **β coefficient** | **SE** | **P value** |
| Age | 0.024 | 0.0.35 | 0.494 | 0.059 | 0.025 | 0.019 † |
| Race | -0.090 | 0.532 | 0.866 | -0.585 | 0.475 | 0.219 |
| Education (y) | -0.066 | 0.059 | 0.266 | -0.056 | 0.055 | 0.311 |
| APOE e4 + | 0.778 | 0.332 | 0.019 † | 0.153 | 0.314 | 0.627 |
| FHD | -0.218 | 0.334 | 0.514 | -0.130 | 0.285 | 0.649 |
| Systolic blood pressure | 0.004 | 0.008 | 0.572 | 0.002 | 0.009 | 0.795 |
| Diastolic blood pressure | 0.021 | 0.017 | 0.220 | -0.005 | 0.014 | 0.710 |
| MMSE | -0.282 | 0.094 | 0.003 † | -0.306 | 0.063 | 0.000 † |
| CDR | 2.950 | 0.483 | 0.000 † | 2.721 | 0.358 | 0.000 † |

† Significant difference between the 2 groups ($p < 0.05$).

Key: FHD, family history of dementia; MMSE, the Mini-Mental State Examination score; CDR, the Clinical Dementia Rating score.

# Appendix 4: Multivariable Cox proportional hazards regression coefficients (adjusted for age) for the risk of converting to MCI

**eTable 3a. Multivariable Cox proportional hazards regression coefficients (adjusted for age) for the risk of converting to MCI in female subgroup.**

| Risk factors | β coefficient | SE | p value | HR | 95.0% CI of HR |
|---|---|---|---|---|---|
| Age | 0.021 | 0.041 | 0.612 | 1.021 | 0.942--1.107 |
| APOE e4 + | 0.207 | 0.342 | 0.545 | 1.230 | 0.629--2.405 |
| MMSE | -0.039 | 0.100 | 0.698 | 0.962 | 0.791--1.170 |
| CDR | 2.857 | 0.499 | 0.000† | 17.415 | 6.545--46.339 |

$S_0(12)$ = 0.929 (average 12-year survival).

P value of the overall model: P < 0.000.

β values are expressed per 1 unit increase for continuous variables and for the condition present in dichotomous variables.

Key: MMSE,the Mini-Mental State Examination score; CDR,the Clinical Dementia Rating score.

**eTable 3b. Multivariable Cox proportional hazards regression coefficients (adjusted for age) for the risk of converting to MCI in male subgroup.**

| Risk factors | β coefficient | SE | p value | HR | 95.0% CI of HR |
|---|---|---|---|---|---|
| Age | 0.028 | 0.027 | 0.291 | 1.029 | 0.976--1.084 |
| APOE e4 + | 0.388 | 0.321 | 0.227 | 1.475 | 0.786--2.768 |
| MMSE | -0.196 | 0.067 | 0.003† | 0.822 | 0.721--0.937 |
| CDR | 2.600 | 0.367 | 0.000† | 13.461 | 6.563--27.610 |

$S_0(12)$ = 0.937 (average 12-year survival).

P value of the overall model: P < 0.000.

β values are expressed per 1 unit increase for continuous variables and for the condition present in dichotomous variables.

Key: MMSE,the Mini-Mental State Examination score; CDR,the Clinical Dementia Rating score.

# Appendix 5: Workflow of selected participants in HABS

**eFigure 1. Workflow of selected participants in HABS.**

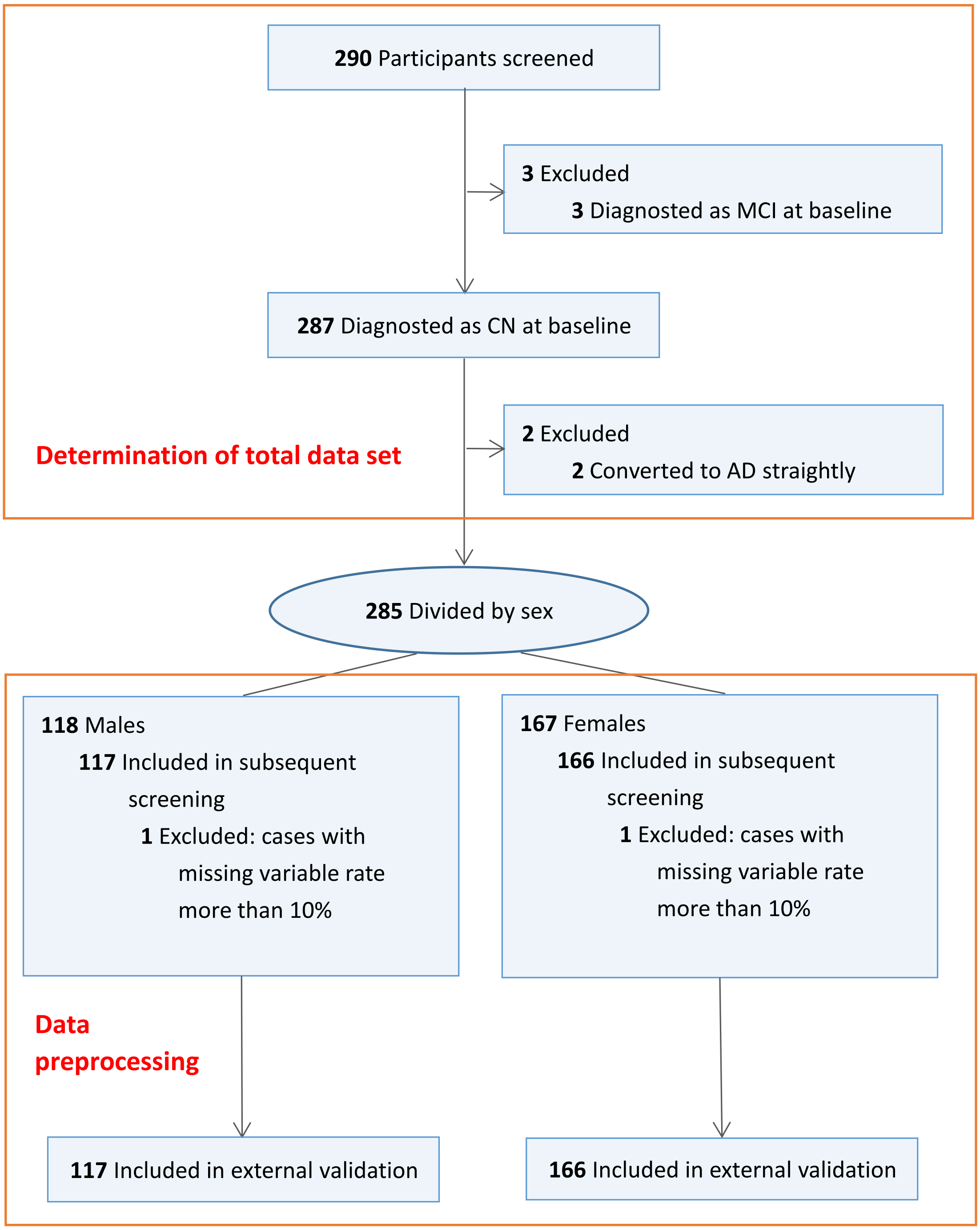

Supplement: Supplementary file 1 [file Data_Sheet_1.PDF]
